# Climate Change and Nursing Care in Major Chronic Diseases: A Scoping Review of Nursing Perspectives and Roles

**DOI:** 10.3390/healthcare14183044

**Published:** 2026-09-16

**Authors:** Sibel Yolcu

**Affiliations:** Department of Internal Medicine Nursing, Atlas University Nursing, Kâğıthane, 34408 Istanbul, Turkey; sibel.yolcu@atlas.edu.tr; Tel.: +90-054-1557-9808

**Keywords:** climate change, chronic disease, nursing care, scoping review

## Abstract

**Background/Objectives:** Climate change produces more pronounced health outcomes in individuals with chronic disease, and nurses are strategically positioned to recognise, prevent, and manage these effects; however, the scope of this relationship within the nursing discipline remains unclear. This review aimed to map the literature on the impacts of climate change on chronic diseases and the associated nursing care, roles, and interventions. **Methods:** Following the Arksey and O’Malley framework and PRISMA-ScR guidance, international (PubMed/MEDLINE, CINAHL, Scopus, Web of Science, Cochrane Library) and Turkish (TR Dizin, DergiPark, YÖK) databases were searched without date restriction between 2 and 22 June 2026. Of 957 records identified, 721 remained after removing 236 duplicates on import; a further 36 duplicates were identified and removed during Rayyan screening, leaving 685 records for title/abstract screening; following full-text screening, 23 sources were included. **Results:** Most sources were published between 2023 and 2026 (73.9%) and were narrative reviews (43.5%); only four studies used a randomised or cohort design (two RCTs, one baseline-characteristics report from an RCT cohort, and one prospective cohort study), alongside one pilot cohort study. Respiratory and multisystem/multimorbid conditions were most common (*n* = 6 each, 26.1%), with general/multiple climate framing (*n* = 9, 39.1%) and extreme heat (*n* = 6, 26.1%) the predominant exposures. Sources came from 10 countries, led by the United States, Türkiye, and Australia, with nurse-affiliated authorship in a majority of sources (65.2%). Patient/family education (73.9%) and environmental risk assessment (65.2%) were the leading nursing roles; telehealth, curriculum integration, and the researcher role were each represented in ≤17.4% of sources. **Conclusions:** Research on the climate change–chronic disease relationship from a nursing perspective remains limited and largely descriptive, reflecting preliminary evidence of nurse-level knowledge gaps that warrants confirmation in larger samples, a weak experimental evidence base, and evidence not yet translated into disease- and season-specific protocols. Quantitative and intervention research is needed in underrepresented categories within this review’s defined scope, such as metabolic disease, alongside stronger nurse-led advocacy, policy, and research roles; because mental health and maternal/newborn outcomes were not comprehensively targeted by the search strategy, whether these represent genuine evidence gaps rather than areas simply outside this review’s scope remains to be established by future, broader-scope reviews.

## 1. Introduction

Climate change is recognised as one of the most serious global health threats of the twenty-first century. Rising greenhouse gas emissions affect human health both directly and indirectly through multiple pathways, including extreme heat and heatwaves, increased levels of air pollution, extreme weather events such as floods, droughts, and wildfires, and changes in the geographical distribution of vector-borne diseases. These effects are particularly pronounced among individuals with chronic diseases; impaired thermoregulation, interactions between medications and environmental conditions, and underlying physiological vulnerability render these populations disproportionately susceptible to climate-related health risks.

For the purposes of this review, a chronic disease is defined, following the World Health Organization and the U.S. Centers for Disease Control and Prevention, as a condition of long duration generally lasting one year or more that results from a combination of genetic, physiological, environmental, and behavioural factors, follows a generally slow course, and requires ongoing medical attention, limits activities of daily living, or both [1,2]. Chronic diseases share several defining characteristics that distinguish them from acute conditions: they are of long duration and rarely resolve completely; they typically follow a gradual, progressive course rather than an acute onset; their aetiology is multifactorial, reflecting the interaction of genetic predisposition, physiological changes, environmental exposures, and behavioural risk factors rather than a single causal agent; and their management is continuous rather than episodic, commonly extending across multiple organ systems, care settings, and members of the healthcare team over the patient’s lifetime. Unlike acute illness, a chronic disease is typically not curable but only managed, leaving affected individuals with a sustained physiological and treatment burden that is directly relevant to climate vulnerability: it reduces the homeostatic reserve available to buffer additional environmental stress (e.g., extreme heat or cold); it relies on continuous pharmacological or device-based management that can itself interact adversely with environmental exposures (e.g., diuretic or ACE-inhibitor effects under heat stress; Section 4.2); and it requires ongoing patient self-management, which is precisely the domain in which nursing care is concentrated (Section 3.7). The four disease categories targeted by this review’s search strategy—chronic obstructive pulmonary disease (COPD)/chronic respiratory disease, diabetes mellitus, cardiovascular disease (including heart failure), and chronic kidney disease, together with hypertension—were selected because each meets this definition and each has an established, climate-sensitive pathophysiology reported in the wider literature, including heat- and pollution-related exacerbation of airway disease, thermoregulatory and glycaemic effects on diabetes, cardiovascular strain under thermal extremes, and heat- or dehydration-related renal injury.

Nurses are strategically positioned to recognise, prevent, and manage the health effects of climate change: as the largest group of healthcare professionals worldwide, they provide direct, continuous, and trusted contact with patients across acute, primary, and community-care settings. In chronic disease care specifically, nurses are typically responsible for patient and family education (e.g., heat-action planning, inhaler technique, self-management support), environmental and vulnerability risk assessment, care coordination and case management, disaster and emergency preparedness, and, increasingly, advocacy for climate-resilient and sustainable models of care roles substantiated empirically by the thematic findings of this review. Despite this central role, the position and scope of research addressing the relationship between climate change and chronic diseases within the nursing discipline remain unclear. A bibliometric analysis conducted by Kes [3] revealed that only three of the 713 articles published on climate change and chronic diseases were directly related to nursing, highlighting a substantial research gap. This gap is corroborated by the wider, and growing, body of international literature on the state of nursing scholarship in this area more broadly: a 2024 mixed-methods systematic review found that the evidence base linking climate change to patient health outcomes remains fragmented and unevenly distributed across disease groups and regions [4], while a companion scoping review of nursing competencies identified that formal “eco-nursing” competencies for climate-related care are still largely undeveloped and inconsistently defined across nursing curricula and practice standards [5]. Taken together, these sources indicate not merely a numerical scarcity of nursing-specific publications on this topic, but a broader lack of synthesis, standardisation, and disease-specific translation of the available evidence: without a clear map of the existing evidence, nurse educators, clinicians, and policymakers cannot identify where knowledge is sufficient to guide practice, where it is only emerging, and where it is effectively absent.

Given this uncertainty, a scoping review rather than a systematic review or meta-analysis is the appropriate design for this stage of inquiry. Scoping reviews are recommended specifically for mapping the extent, range, and nature of an emerging and heterogeneous body of evidence, clarifying how key concepts are used across the literature, and identifying gaps, rather than for pooling effect estimates from a narrowly defined set of comparable studies [6,7,8]. Because the climate change–chronic disease–nursing intersection spans highly heterogeneous evidence types from bibliometric analyses and narrative reviews to a small number of RCTs and cohort studies (Section 3.5) across varied disease categories and climate-related hazards, a systematic review with quantitative pooling is neither feasible nor appropriate at this stage; a scoping review instead allows the full breadth of available evidence to be mapped, which is a necessary precursor to any future, more narrowly focused systematic review. This scoping review was therefore designed to map the nature, scope, and distribution of the existing evidence base; identify the chronic disease categories and climate-related risks addressed in the literature; examine how nursing care is positioned within these sources; and determine priority gaps for future research.

### Aim of the Review

This review aims to systematically map, in accordance with the PRISMA-ScR guideline, the existing literature addressing the relationship between climate change and major chronic non-communicable diseases (COPD/chronic respiratory disease, diabetes mellitus, cardiovascular disease including heart failure, and chronic kidney disease, as reflected in the search strategy in Table 1) and the role of nursing care within this context. The review’s climate-hazard scope is similarly bounded by its search strategy (Table 1) to extreme heat, air pollution/greenhouse gas emissions, vector-borne disease, and extreme cold; other hazards documented in the wider climate–health literature, including wildfire smoke, flooding, and other extreme weather events, were not targeted by the search terms and are not addressed here. This is a deliberate scope boundary of the present review, not an assumption that these hazards or disease categories are unrelated to nursing and chronic disease (see Section 5). The title deliberately refers to nursing “perspectives and roles” rather than to nursing care alone: as Section 3.7 details, nursing relevance across the included sources ranges from direct evaluation of a nursing intervention, practice, or competency to indirect representation through authorship, study sample, or journal context, and the title is intended to reflect that fuller spectrum rather than imply that every included source directly studied nursing care. Accordingly, using the Population–Concept–Context (PCC) framework detailed in Table 2, the review sought to answer the following research questions:

- (Population) Which chronic disease categories are most frequently addressed in the context of climate change?
- (Concept) Which types of climate-related risks such as extreme heat, air pollution, and greenhouse gas emissions predominate in the literature?
- (Context) In what ways is nursing care represented in these sources in terms of authorship, study population, interventions, and journal context?
- (Concept) What is the distribution of study designs used in the existing evidence base?
- (Concept/Context) What research gaps remain in the literature?

## 2. Methods

### 2.1. Study Design

Given the exploratory and descriptive nature of the research aim, this study adopted a scoping review design to map the effects of climate change on chronic diseases and examine how the scope of nursing care, roles, and interventions is positioned within this body of research. Scoping reviews address broad research objectives, identify areas requiring further synthesis, and map the available evidence in fields that remain underexplored. The methodological process followed the frameworks proposed by Arksey and O’Malley [6], Levac et al. [7], and the Joanna Briggs Institute (JBI). Consistent with the nature of a scoping review, no formal assessment of methodological quality or risk of bias was conducted for the included sources; instead, each source is reported by study design only, as a descriptive characteristic rather than a ranking of evidence quality (see Section 2.6).

**Protocol and Registration**

The scoping review protocol was retrospectively registered with the Open Science Framework (OSF) after completion of the review process (osf.io/j7bfy). The reporting of this review was guided by the Preferred Reporting Items for Systematic Reviews and Meta-Analyses Extension for Scoping Reviews (PRISMA-ScR) [9], and the completed PRISMA 2020 checklist and flow diagram are provided as Supplementary Materials. The protocol is accessible at the OSF registration link above, and no amendments were made to the information provided at registration.

### 2.2. Identification of Relevant Sources

**Research Strategy**

The academic literature search was conducted by the author using the following databases:

- International databases: PubMed/MEDLINE, CINAHL, Scopus, Web of Science, and the Cochrane Library.
- National Turkish databases: TR Dizin, DergiPark, and the Council of Higher Education (YÖK) National Thesis Center.

No restrictions were imposed on publication date. The comprehensive search was conducted between 2 and 22 June 2026 (Table 1).

The principal search terms were determined by examining relevant keywords used in previous literature reviews and drawing on the author’s clinical and academic expertise. The search strategy was based on three concept blocks combined using Boolean operators: (A) climate/temperature exposure, (B) chronic disease (restricted, per Section Aim of the Review and Table 2, to COPD/chronic respiratory disease, diabetes, cardiovascular disease including heart failure, and chronic kidney disease), and (C) nursing. Within block C, the terms “patient education” and “self-management” were included alongside “nurse”/”nursing” because supporting patients’ self-management of chronic disease adjusting behaviour and treatment in response to environmental triggers such as heat or poor air quality is one of the core nursing functions named under the Concept criterion in Table 2, and much of the applied nursing literature on chronic disease indexes itself under “self-management” rather than “nursing care” specifically. All retrieved records were imported into the Zotero (Digital Scholar, Vienna, VA, USA; version 9.0.6) reference management software (*n* = 957). After duplicate removal, 721 records remained and were screened using Rayyan software (Rayyan Systems Inc., Cambridge, MA, USA; accessed via new.rayyan.ai) [10] (Table 1).

### 2.3. Eligibility Criteria

Sources published in English or Turkish were considered eligible, with no date restrictions, in accordance with the Population–Concept–Context (PCC) framework presented in Table 2. For empirical sources (i.e., those reporting original data collection or analysis), records were excluded as having “unclear methods” when, even after consulting the full text, no study design was stated, no description was given of how participants or data were selected, and, for quantitative studies, no analytic method was reported. This criterion was not applied to narrative reviews, position statements, or other non-empirical evidence sources, which were assessed only against criteria E1–E3 and E5 below. Case reports and case-report-based reviews were treated as empirical sources for the purposes of this criterion, since they report data drawn from an identified patient case, and were assessed against the “unclear methods” test on that basis.

Based on the recommendations of the Joanna Briggs Institute methodology for scoping reviews [8], the eligibility criteria were structured according to the Population–Concept–Context (PCC) framework and are presented in Table 2.

During full-text assessment, sources were excluded for one of the following reasons:

- E1—No climate/heat dimension: No explicit or causal link was established between the topic under investigation and climate change or a climate-related hazard.
- E2—No chronic disease focus: The study did not focus on a named, specific chronic disease or condition.
- E3—No nursing/care dimension: A nursing perspective was absent from the authorship team, study population, and content.
- E4—Ineligible publication type: The publication was a conference abstract, letter, or editorial without substantive content, or a duplicate/superseded version of an already-included record. Narrative reviews, position statements, and other non-empirical sources of evidence were eligible provided they met criteria E1–E3 and E5; consistent with scoping review conventions, these are referred to as “sources of evidence” rather than “studies” where the distinction matters (see Section 2.1).
- E5—Ineligible population: The study population was inconsistent with the target population defined in the PCC framework.

### 2.4. Record Selection Process

**Figure 1:**
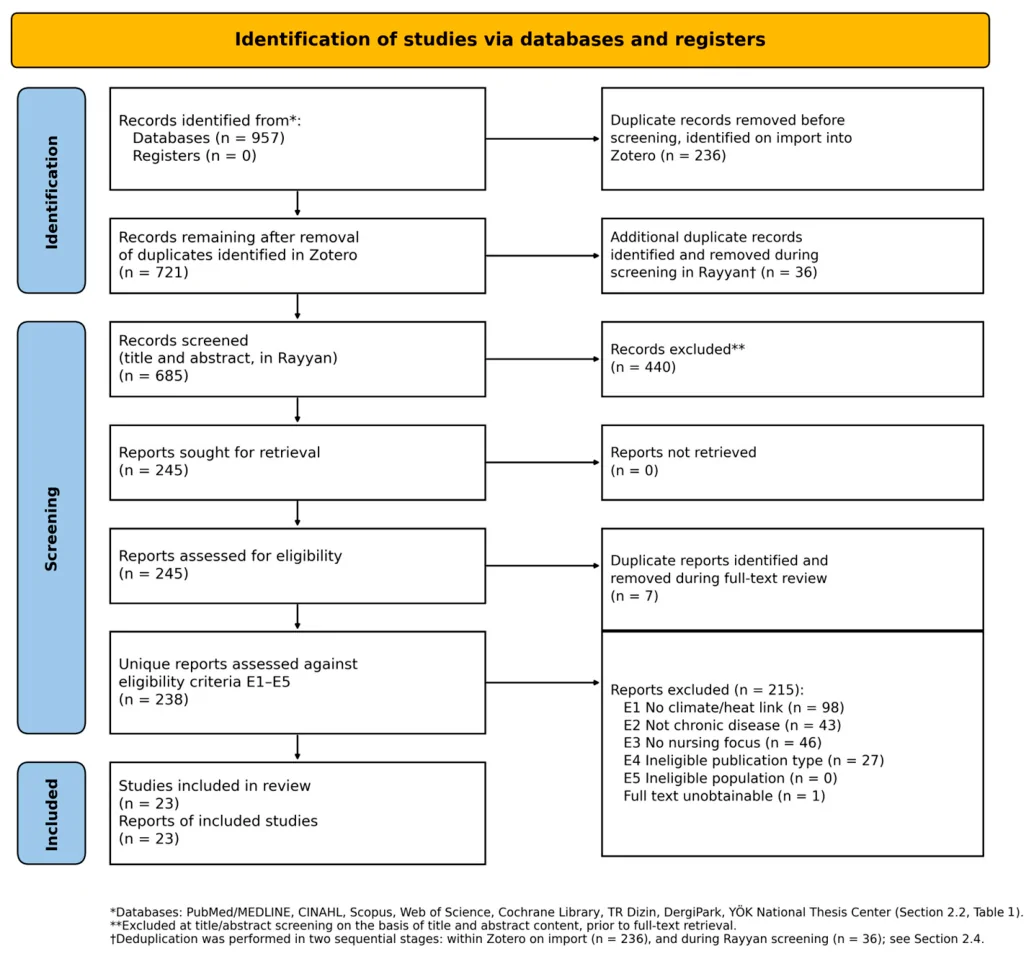
PRISMA 2020 flow diagram of the record selection process, adapted for this scoping review (PRISMA-ScR) in accordance with the official PRISMA 2020 template.

The record selection process is presented in Figure 1 as a PRISMA-ScR flow diagram, which follows PRISMA 2020 terminology: records at identification and title/abstract screening, and reports from full-text retrieval onward. Following import into Zotero, 236 duplicate records were removed from the 957 records retrieved, leaving 721 unique records (Section 2.2). During subsequent screening in Rayyan, 36 additional duplicate records were identified and removed, leaving 685 records for title and abstract screening. At this stage, 440 records were excluded, and reports for the remaining 245 records were sought for retrieval; all 245 reports were successfully retrieved. During full-text assessment, seven additional duplicate reports were identified through manual review and removed, leaving 238 unique reports assessed individually against eligibility criteria E1–E5. Of these, 214 reports were excluded according to criteria E1–E5. One further report, for which the full text could not be obtained despite contacting the authors, was subsequently excluded on sensitivity grounds because abstract-level information alone was judged insufficient for reliable data extraction (see Section 2.6 and Section 5). This resulted in the inclusion of 23 sources in the final review. Title/abstract and full-text screening were conducted by a single reviewer (S.Y.). Because this review was conducted by a sole author, independent duplicate screening and data extraction by a second reviewer were not performed. To reduce the risk of selection and coding error, the standardised extraction form (Supplementary Materials, Table S2) was piloted on five (approximately 22%) of the 23 included sources before being applied to the full sample; following the pilot, no changes to the form were judged necessary. Three decision rules, established prospectively and applied consistently throughout screening and extraction, addressed the borderline cases encountered most frequently: (i) sources addressing more than one chronic disease category were coded to all applicable categories rather than to a single primary category; (ii) sources describing a climate-related hazard only in general terms (e.g., “extreme weather”, without further specification) were coded under the general/multiple climate-hazard category rather than excluded under E1; and (iii) sources in which a nursing perspective was present only through journal scope, with no named nursing author, role, or content, were excluded under E3. These rules are reproduced in the Supplementary Materials (see Section 5).

### 2.5. Data Extraction

Data extraction was performed by a single reviewer (S.Y.) using a standardised, pre-piloted extraction form; as this review was conducted by a sole author, data were not independently extracted or verified by a second reviewer. For each included study, the following information was recorded using a standardised data extraction form: author(s), year of publication, title, journal, country, language, study design, study aim, sample population and size, age group, sex distribution, how climate change was addressed in the study (central focus, contextual motivation, or subsection), type of climate-related risk, chronic disease diagnosis and category, nursing care component, nursing role/intervention, principal findings, nursing-related findings/recommendations, study design classification, and notes on methodological limitations. The complete data extraction form is provided in the Supplementary Materials (Table S2). The free-text “nursing role/intervention” field of the extraction form was subsequently used, at the synthesis stage (Section 2.7), to derive the thematic categories reported in Section 3.7: candidate themes were generated inductively by the single author (S.Y.) through iterative comparison of this field across all included sources, then consolidated into 12 domains, each accompanied by an explicit operational definition used to adjudicate boundary cases—for example, content was coded to “Patient/Family Education and Counselling” when it described one-off instruction or counselling and to “Self-Management Support” when it instead described support for the patient’s own ongoing behavioural adjustment; a single source could be coded to more than one domain when it addressed multiple nursing functions.

### 2.6. Reporting of Study Design

Consistent with scoping review methodology, no formal risk-of-bias or quality appraisal was conducted, and this review does not assign or imply a summary judgement of evidence “quality.” To orient the reader, each included source is instead categorised solely by study design (e.g., randomised controlled trial, cohort study, cross-sectional study/survey, qualitative study, narrative review/expert opinion, bibliometric analysis). This is reported as a descriptive characteristic only; it is not a ranking of evidence quality, since study quality varies substantially within any single design category. Design labels were assigned from the terminology used by each source’s own authors (e.g., a source self-described as a pilot study was labelled “Pilot Cohort Study” rather than “Cohort Study”, and a source reporting only the baseline characteristics of an RCT cohort, without outcome data, was labelled “RCT (Baseline Characteristics Report)” rather than “Randomised Controlled Trial”); where a source did not self-identify its design, the label was assigned by the single author (S.Y.) based on the study features described in Section 2.1 of the JBI/Arksey-and-O’Malley framework, consistent with the absence of a formal risk-of-bias tool noted above.

### 2.7. Data Synthesis

The extracted data were summarised using descriptive statistics, including frequencies and percentages, and presented through a narrative synthesis. The findings were organised according to disease category, type of climate-related risk, study design, country, and year of publication.

## 3. Results

### 3.1. Study Selection Process

Of the 957 records initially identified, 23 sources were included in the review following the selection process described in Section 2.4. Details of the record selection process are presented in Figure 1.

### 3.2. Characteristics of the Included Sources

The 23 included sources were published between 2013 and 2026, with the majority (*n* = 17, 73.9%) published between 2023 and 2026, indicating a marked increase in research interest in this field in recent years. The 23 included sources originated from 10 countries in total, most frequently the United States (*n* = 7), Türkiye (*n* = 4), and Australia (*n* = 3). Methodologically, narrative reviews/position papers predominated (*n* = 10). Only two randomised controlled trials, one baseline-characteristics report from an RCT cohort, and one cohort study were identified, alongside one pilot cohort study, indicating that the production of experimental and analytical evidence in this field remains limited. The complete list and characteristics of the included sources are presented in Table 3.

### 3.3. Chronic Disease Categories

The disease categories most frequently addressed in the included sources were respiratory diseases (*n* = 6, 26.1%; primarily asthma and chronic obstructive pulmonary disease [COPD]) and multisystem/multimorbid conditions (*n* = 6, 26.1%). These were followed by cardiovascular diseases (*n* = 5, 21.7%). Kidney diseases (*n* = 2), mental health conditions (*n* = 1), metabolic diseases/diabetes (*n* = 1), rheumatological/infectious conditions (*n* = 1), and women’s health/neonatal outcomes (*n* = 1; gestational diabetes mellitus and pre-eclampsia, eligible under the pregnancy-related manifestation criterion specified in Table 2) were represented less frequently. The distribution is presented in Table 4.

### 3.4. Types of Climate-Related Risk

A substantial proportion of the sources (*n* = 9, 39.1%) addressed climate change within a general/multidimensional framework rather than focusing on a single climate-related hazard. Extreme heat/heatwaves were the most frequently examined specific climate-related risk (*n* = 6, 26.1%), followed by the greenhouse gas emissions/carbon footprint perspective (*n* = 3, 13.0%). Air pollution, extreme cold, and vector-borne diseases were addressed in a more limited number of sources. The distribution is presented in Table 5.

### 3.5. Study Designs

From a methodological perspective, nearly half of the included sources (*n* = 10, 43.5%) were narrative reviews. Experimental and analytical designs were limited: two randomised controlled trials (RCTs), one report of baseline characteristics from an RCT cohort, one prospective cohort study, and one pilot cohort study were identified. In addition, four cross-sectional/survey studies, two case reports combined with reviews, one qualitative study, and one bibliometric analysis were included. One further record, for which the full text could not be obtained, was excluded on sensitivity grounds rather than retained at the abstract level (see Section 2.4 and Section 5). Study designs are summarised descriptively in Table 6; consistent with Section 2.6, this classification is not a ranking of evidence quality.

### 3.6. Geographical Distribution

The included sources originated from 10 countries, with the largest contributions from the United States (*n* = 7, 30.4%), Türkiye (*n* = 4, 17.4%), and Australia (*n* = 3, 13.0%). This distribution is presented in Table 7.

### 3.7. Position of Nursing Care Within the Included Sources

Of the 23 included sources, 15 (65.2%) had authorship teams composed wholly or partly of nurses, 11 (47.8%) were published in nursing journals, and four (17.4%) included nurses in their study samples. These categories were not mutually exclusive, and some sources were represented in more than one category. Nursing relevance conferred solely through authorship or journal context, however, does not necessarily mean a source examined nursing care directly: only a subset of the 23 sources—those explicitly evaluating a nursing intervention, role, or competency as part of the study aim (e.g., [17,19,22,23,24,25,27,29]) assessed nursing care directly, whereas the remainder addressed it indirectly, through authorship, journal context, or general clinical recommendations.

The thematic analysis of “Nursing Roles and Interventions” showed that nursing practices at the intersection of climate change and chronic disease were concentrated across 12 functional domains (Table 8). Categories were derived inductively: the free-text “nursing role/intervention” field recorded for each source in the data-extraction form (Supplementary Materials, Table S2) was reviewed by the single author (S.Y.) and grouped into candidate themes through iterative comparison across sources, then consolidated into the 12 domains in Table 8; a source could be coded to more than one domain when it addressed multiple nursing functions, so category totals sum to more than 23. The most prevalent theme was patient/family education and counselling (73.9%), encompassing a broad range of activities, from heat action plans and inhaler technique training to air-quality alerts and disaster preparedness. Environmental risk assessment/screening ranked second (65.2%), with nurses conducting multidimensional assessments of factors such as occupational exposure, air pollution, heat stress, and socioeconomic vulnerability. Advocacy ranked third (39.1%), reflecting nurses’ efforts to promote environmental justice, sustainable/green prescribing, and climate-change mitigation policies. Policy development and sustainable/green practices (30.4%), particularly reducing carbon footprints, selecting inhalers with a low global warming potential (GWP), and implementing green nephrology practices, indicated that nursing practice is becoming increasingly integrated with institutional sustainability.

Less frequently identified but clinically substantive themes included disaster/emergency preparedness (26.1%), care coordination/case management (21.7%), and evidence generation/the researcher role (17.4%). The RESILIENCE nurse model described by Stewart et al. [28,30] is particularly noteworthy as a home visit-based, multicomponent, nurse-coordinated care approach (8.7%); because both publications derive from the same RESILIENCE trial cohort, this reflects one underlying study rather than two independent sources of evidence. In sources such as those by Dönmez and Kurt [27] and Padilla and Sabol [32], the nurse’s multifaceted professional identity, based on the frameworks of the International Council of Nurses (ICN) and the American Nurses Association (ANA), was explicitly emphasised and encompassed the roles of educator, advocate, change agent, leader, caregiver, and supervisor (8.7%).

The findings indicate that, in the context of climate change, nursing care predominantly fulfils educational, preventive, and early-detection functions. However, policy-level advocacy and systemic sustainability initiatives have also emerged as a distinct subtheme. In contrast, telehealth/surveillance-based monitoring models and proposals for formal curriculum integration remain relatively new areas addressed in only a limited number of sources (13.0% each).

### 3.8. Cross-Cutting Synthesis: Relating Disease, Hazard, and Nursing Role

Read together, the disease-category (Section 3.3), climate-hazard (Section 3.4), and nursing-role (Section 3.7) distributions reported above are not independent: the same small set of sources accounts for most of the overlap between them. The only two sources providing experimental evidence of a nursing intervention (Jehn et al. [25]; Stewart et al. [28]) both target a single disease–hazard pairing respiratory disease and cardiovascular/multimorbid disease, respectively, under extreme-heat exposure and both instantiate a narrow set of nursing roles (telemonitoring/care coordination and home-visit-based case management). Outside this narrow evidence core, the disease categories that are best represented overall (respiratory, cardiovascular, multisystem; Table 4) are also the categories for which the widest range of nursing roles is reported (patient education, environmental risk assessment, advocacy, policy development; Table 8) but this breadth is achieved almost entirely through narrative reviews and cross-sectional surveys (Table 6) rather than through sources that test whether these roles change patient outcomes. Conversely, the disease categories that are least represented (mental health, metabolic disease, women’s/neonatal health; Table 4) are each covered by only a single source, so no meaningful disease–hazard–role pattern can be drawn for these categories at all. This absence of pattern is itself a principal finding of this review, not a gap in reporting: it identifies precisely where evidence is too sparse to guide practice (Section 4 and Section 6). In short, the apparent breadth of the nursing-role findings (12 domains, Table 8) is concentrated in a small number of well-covered disease–hazard combinations and rests overwhelmingly on descriptive rather than intervention evidence; this cross-cutting pattern, rather than any single distribution taken in isolation, is what this review identifies as the priority target for future nurse-led intervention research (Section 6).

## 4. Discussion

### 4.1. Overview of the Evidence Base

The findings of this scoping review indicate that the literature addressing the relationship between climate change and chronic diseases from a nursing care perspective has expanded rapidly in recent years, yet the field remains dominated by descriptive studies and reviews. The publication of 73.9% of the included sources within the past four years (2023–2026) suggests that academic interest in this topic has gained momentum. This finding is consistent with the overall upward trend observed in Kes’s [3] bibliometric analysis of 713 articles. Throughout this Discussion, findings are drawn from the 23 sources included in this review’s synthesis. Kes [3] is one of these 23 included sources (Table 3) and is referenced here, alongside the review’s own findings, because its bibliometric analysis of the wider literature independently corroborates the research gap this review maps; the sole exception is the 2025 Lancet Countdown report [33] (Section 4.6), which is explicitly identified as external context, is not among the 23 included sources, and is cited only to situate the review’s findings within the broader literature.

The predominance of respiratory and cardiovascular diseases in the literature may be explained by the more direct pathophysiological sensitivity of these disease groups to climate- and weather-related variables, including temperature, air pollution, and pollen exposure. In contrast, the limited representation of mental health conditions, including climate anxiety and depression, and metabolic diseases such as diabetes suggests that these remain emerging areas of research that are likely to expand in the future, as indicated by recent sources such as those by Mehta et al. [21] and Padilla and Sabol [32].

### 4.2. Underrepresented Disease Categories and the Limits of a Small Evidence Base

The limited representation of the mental health and women’s health/neonatal categories can be better understood by examining the two sources that served as the sole representatives of these categories. The study by Mehta et al. [21] was one of only four studies in this review to use a randomised or cohort design. It was conducted within the Nurses’ Health Study II cohort of 39,339 nurses in the United States between 2001 and 2019 and demonstrated that each 4.9 °C increase in mean annual temperature was significantly associated with an increased incidence of clinician-diagnosed depression (fully adjusted HR, 1.06; 95% CI, 1.02–1.08). This study was the first prospective cohort investigation to demonstrate an association between long-term temperature exposure, defined by mean annual temperature, and the incidence of depression. The authors emphasised that climate–health messaging has not yet been systematically integrated into mental health surveillance. This observation aligns with the finding that the “monitoring/surveillance/telehealth” theme was represented in only 13.0% of the included sources (Table 8). The review by Dönmez and Kurt [27], the sole study representing the women’s health/neonatal category, was eligible under the Population criterion specified in Table 2: it reports that exposure to high temperatures and air pollution was associated with gestational diabetes mellitus and pre-eclampsia pregnancy-specific manifestations of the diabetes mellitus and cardiovascular/hypertensive disease categories targeted by this review alongside preterm birth and low birth weight, which are reported here as accompanying outcomes rather than as the basis for its inclusion. As a narrative review, this source addressed nursing practice through general roles based on the ICN framework rather than through a specific intervention. Together, these two sources demonstrate that the strength of the underlying evidence is not homogeneous, even within underrepresented categories.

Beyond this categorical distribution, two sources illustrate the opposing extremes of climate exposure at the individual level: Hodges and Al Hodges [16] reported a case of heat-related dehydration and acute kidney injury in a patient with type 2 diabetes and hypertension, worsened by an ACE inhibitor/diuretic combination, while Gumabay et al. [17], in a qualitative study of 30 Filipino patients with coronary artery disease, reported that prolonged cold exposure triggered angina through sympathetic activation and vasoconstriction. Together, these sources indicate that both heat and cold can exacerbate chronic disease through distinct pathophysiological pathways, challenging the unipolar, heat-only framing that dominates much of the reviewed literature (Table 5). Although a single case narrative and a small qualitative study are reported here descriptively rather than ranked against experimental evidence (Section 2.6), both yielded a concrete nursing measure—heat action plans and clothing/heating counselling that maps directly onto the patient/family-education theme identified as this review’s most prevalent nursing role (Section 3.7).

### 4.3. The Scarcity of Experimental Evidence

From a methodological perspective, the most striking finding was the scarcity of experimental and analytical designs, particularly randomised controlled trials. Only three of the 23 included publications used a randomised design (two RCTs and one baseline-characteristics report from an RCT cohort), and only one study, Jehn et al. [25], directly tested the effectiveness of a nursing intervention. As also emphasised by Kes [3], this finding confirms a substantial lack of intervention research addressing the climate change–chronic disease relationship within nursing.

This single RCT [25] illustrates what such intervention research can look like: in a German trial of 62 COPD patients randomised to home-based telemonitoring versus standard care, the telemonitoring group had markedly fewer summer-season exacerbations (3 vs. 14; *p* = 0.006) and shorter cumulative hospital stays (34 vs. 97 days), while lung function and walking distance still deteriorated in both groups on high-heat days—evidence that nurse-led telemonitoring can mitigate, but not eliminate, heat-driven exacerbation. Its small, single-centre sample means it cannot by itself supply the evidence base the field needs.

### 4.4. How Directly Do These Sources Examine Nursing Care?

An examination of how nursing care was positioned within the included sources showed that, in a substantial proportion, the nursing perspective was represented indirectly through authorship, study samples, or journal context. However, only a limited number of sources were directly designed and led by nurses and targeted the quality of patient care or clinical outcomes as outcome measures. This finding indicates that the nursing discipline should assume a more visible role in climate health as both a research and practice profession.

Although “patient/family education and counselling” was the most frequently coded nursing role (73.9%), the survey by Chaseling et al. [20] of Australian cardiac rehabilitation clinicians (70% nurses) found this role was not underpinned by a robust evidence base: only 17% believed sufficient information existed on the heat–cardiovascular disease relationship, and clinicians who were aware of medication–heat interactions still reported unresolved guidance on conflicting hydration messages—fluid restriction in heart failure versus general heat-health advice to increase fluid intake. This finding supports this review’s broader conclusion that the quality of evidence underpinning nursing education warrants scrutiny before its quantity.

A similar pattern emerged in Frantzen and Kaifie’s [22] cross-sectional survey of German home-care nursing services: although 90% of participants correctly linked climate change to cardiovascular risk, the same proportion had never received heat-protection training, and over a third felt inadequately prepared to implement protective measures. Read together, Chaseling et al. [20] and Frantzen and Kaifie [22] converge across different clinical contexts on a pattern of “high awareness but limited actionable knowledge,” consistent with the structural knowledge gap this review identifies at the clinician level (Section 4.7).

Frantzen and Kaifie [22] also showed that nurses are affected by this crisis not only as educators but as workers exposed to heat stress themselves, with substantial proportions reporting no additional breaks or appropriate clothing on hot days. Although occupational heat exposure is not itself a chronic-disease outcome, it is not a separate topic here: the same home-care and district nurses who deliver the patient education this review maps (Section 3.7) are themselves working unprotected in the conditions their patients are advised to avoid, representing a direct, if underexplored, link back to this review’s central concern and a research gap warranting a dedicated category in future reviews.

### 4.5. A Case Study in Nurse-Led Intervention Evidence: RESILIENCE

The RESILIENCE trial [28], a pragmatic RCT of a nurse-coordinated, multicomponent home-visit intervention, did not improve its primary outcome overall, but a subgroup analysis found significantly shorter summer-season hospitalisations in the intervention group suggesting the approach may work better aimed at narrower, season- and disease-specific targets than at general heat resilience. The trial’s own baseline-characteristics report [30] offers a plausible reason why: women in the cohort showed significantly greater physiological vulnerability and socioeconomic disadvantage (e.g., living alone) than men, implying that future nurse-led interventions of this kind may need to be stratified by sex-related and socioeconomic risk, not only by disease and season.

### 4.6. Global Context (External Source)

The 2025 Lancet Countdown report [33] cited here only for global context and not among this review’s 23 included sources situates these findings within the wider evidence: heat-related mortality rose 63.2% from 1990–1999 to 2012–2021, and only 64% of medical students worldwide (40% in low-Human Development Index countries) had received climate–health education, suggesting the knowledge gap identified in this review is not specific to nursing. It also echoes the fluid-restriction-versus-heat-advice conflict raised by Chaseling et al. [20], one of this review’s included sources.

### 4.7. Synthesis: A Three-Tiered Problem

Considered together, four sources within this review Chaseling et al. [20], Frantzen and Kaifie [22], and Stewart et al. [28,30] together with the external context provided by Romanello et al. [33] (Section 4.6), are consistent with a tentative three-tiered pattern that warrants testing in larger, more representative samples rather than being generalised to the nursing profession as a whole: (1) preliminary evidence of gaps in knowledge and self-efficacy at the clinician and nurse level; (2) a weak experimental evidence base underpinning that knowledge, particularly at the RCT level; and (3) limited translation of existing evidence into actionable, disease- and season-specific clinical guidelines. These three tiers reinforce one another: a weak evidence base leads to inadequate education, which in turn results in inconsistent clinical practices, such as 20% of clinicians providing no recommendations regarding cooling strategies. This cycle indicates that future research should aim not only to improve knowledge levels but also to strengthen the evidence base through experimental designs and translate the resulting evidence into disease-specific, seasonal clinical protocols.

## 5. Limitations

This scoping review has several limitations.

- Title/abstract and full-text screening, and data extraction, were conducted by a single researcher (S.Y.); agreement was not assessed with a second independent reviewer, which may have increased the risk of subjective judgement in borderline cases. Four concrete measures were taken to minimise this risk of error and subjectivity: (i) a standardised, pre-piloted extraction form (Supplementary Materials, Table S2) was used for every source and was first piloted on five sources (approximately 22% of the final sample) before being applied to the full set, with no changes judged necessary after piloting (Section 2.4); (ii) three decision rules for the most common borderline eligibility and coding situations were specified prospectively, before screening began, and applied consistently throughout (Section 2.4); (iii) the complete data-extraction form, the pre-specified decision rules, and the PRISMA-ScR record-selection counts are reported transparently in the Supplementary Materials, and Section 2.4, so that a second reviewer could audit or repeat the screening and coding process directly; and (iv) the descriptive findings and thematic frequencies reported here are explicitly treated as preliminary and hypothesis-generating rather than definitive, precisely because of this single-reviewer design. Independent duplicate screening by a second reviewer, recommended for future updates, was not feasible within the resources of this single-author study; a future update should quantify inter-rater agreement on eligibility and coding decisions using a validated statistic such as Cohen’s kappa once a second reviewer is available (Section 6).
- As stated in the Section titled Aim of the Review, the search strategy deliberately targeted a bounded set of climate hazards (extreme heat, air pollution/greenhouse gas emissions, vector-borne disease, and extreme cold) and chronic disease categories (COPD/chronic respiratory disease, diabetes, cardiovascular disease including heart failure, chronic kidney disease, and hypertension). Hazards such as wildfire smoke, flooding, and other extreme weather events, and disease categories such as cancer, mental health conditions, and neurological disease, were not searched and are consequently absent from this review; this absence reflects the review’s scope boundary and should not be read as evidence that these hazards or diseases are unrelated to nursing and chronic disease. A broader-scope follow-on review covering additional hazards and disease categories is recommended as future work (Section 6).
- The search was limited to sources published in English and Turkish. Consequently, relevant studies published in Spanish, French, Chinese, or Portuguese, particularly those addressing contexts in the Global South, may have been excluded. Relatedly, despite the inclusion of Turkish-language databases, the large majority of included sources originated from high-income countries. The substantial literature on climate change and chronic disease from Asian and, to a lesser extent, African countries exists in the broader climate–health field; its near-absence here most plausibly reflects the review’s English/Turkish language restriction, its specific disease- and hazard-related search terms (which may not match the indexing conventions used in Asian and African journals), and the database selection (Section 2.2), rather than a genuine absence of relevant regional evidence. Evidence from, and applicability to, health systems in the Global South therefore remains limited in this review, and the recommendations in Section 6 should not be generalised to such settings without further, deliberately regionally targeted research.
- The full text of one record [34] could not be obtained despite attempts to contact the authors. Rather than retaining it at the abstract level only, it was excluded from the final synthesis on sensitivity grounds (final *n* = 23; see Section 2.4), because abstract-level information alone was judged insufficient for reliable data extraction and categorisation.
- Consistent with scoping review methodology, no formal risk-of-bias or evidence-quality appraisal tool was applied or reported. Sources are described only by study design (Section 2.6); this description should not be read as an indicator of methodological quality, which was not formally assessed.
- Because most included sources were descriptive or review-based and demonstrated substantial methodological heterogeneity, meta-analytic pooling of effect sizes was not possible.
- The search was limited to academic databases, and the grey literature, including conference proceedings, institutional reports, and policy documents, was not systematically searched. Finally, the comprehensive search was completed in June 2026.
- Given the rapid growth of this field, additional sources may have been published since the review was completed; the findings therefore represent a time-specific snapshot of the available evidence. One included source [27] centres on a pregnant, rather than general adult, population; it was eligible under the pregnancy-related manifestation criterion specified in the Population component of Table 2, because its climate-related outcomes (gestational diabetes mellitus, pre-eclampsia) are pregnancy-specific instances of the diabetes mellitus and cardiovascular/hypertensive disease categories targeted by this review. Its accompanying neonatal outcomes (preterm birth, low birth weight) are reported descriptively but were not themselves the basis for inclusion.
- The scoping review protocol was registered retrospectively, after completion of the review process, which departs from best-practice recommendations for prospective registration.

## 6. Conclusions and Recommendations

This scoping review systematically mapped 23 sources addressing the relationship between climate change and chronic diseases from a nursing care perspective. The findings demonstrate that research interest in this field increased rapidly, particularly between 2023 and 2026. Nevertheless, the evidence base remains largely descriptive and review-based, with a marked scarcity of experimental and analytical designs, particularly randomised controlled trials. Respiratory, cardiovascular, and multisystem conditions, together with extreme heat and heatwaves, emerged as the predominant categories in the literature. Metabolic disease was, in contrast, represented by only one source within this review’s defined scope, an emerging research gap that warrants cautious interpretation given this small evidence base; mental health and women’s health/neonatal outcomes were not comprehensively targeted by the search strategy (Section Aim of the Review), so their apparent underrepresentation here should not be read as an established gap in the literature, but rather flagged as a candidate direction for future, broader-scope reviews (Section 5). An examination of how nursing care was positioned within these sources showed that patient/family education and environmental risk assessment were the predominant themes, although these were often represented only indirectly, through authorship or journal context, rather than through direct evaluation of nursing practice. In the smaller subset of sources that assessed it directly, this educational role was often not supported by a robust evidence base [20,22], while systemic roles such as advocacy, policy development, and telehealth/surveillance remained comparatively underdeveloped. Based on this limited evidence base, this review suggests the field may face a three-tiered problem: (1) preliminary evidence of knowledge and self-efficacy gaps at the clinician and nurse level; (2) a weak experimental evidence base underpinning that knowledge; and (3) a failure to translate existing evidence into actionable, disease- and season-specific clinical protocols. These three tiers were consistently observed both in the underrepresented categories identified in this review [21,27] and at the opposing extremes of climate exposure [16,17], although confirmation in larger, more representative samples is needed. Because the evidence base underlying the following recommendations is drawn overwhelmingly from high-income health systems (Section 5), these recommendations should not be read as universally applicable: their translation into practice will necessarily depend on locally available resources, infrastructure, and healthcare organisation, and should be adapted rather than adopted wholesale in lower-resource and Global South settings.

- The number of nurse-led intervention studies, particularly those employing RCT designs, should be increased. Jehn et al. [25] and the RESILIENCE study by Stewart et al. [28] provide valuable examples of how such interventions may be designed.
- Quantitative and intervention research should be prioritised in underrepresented categories that fell within this review’s defined scope, including metabolic diseases and rheumatological/infectious diseases; a broader-scope review is needed before mental health and women’s health/neonatal outcomes can be confirmed as genuine gaps rather than areas outside the present search strategy (Section 5).
- Future research should stratify interventions not only by chronic disease diagnosis but also by season [28] and by sex-related biobehavioural and socioeconomic patterns of vulnerability [30].
- Research that considers nurses not only as care providers but also as workers exposed to heat stress should be expanded [22].
- In future updates, the use of two independent reviewers with inter-rater agreement on eligibility and coding decisions quantified using a validated statistic, such as Cohen’s kappa together with a formal evidence-level or quality-appraisal tool, would help address the methodological limitations of this review.
- Climate change and its health effects should be systematically integrated into undergraduate and postgraduate nursing curricula [18,35].
- Disease-specific and seasonally adapted clinical protocols and decision-support tools should be developed to resolve conflicts between chronic disease management, such as fluid restriction in heart failure, and general heat-health recommendations, including increased fluid intake [20,33].
- Heat action plans and community health nursing-based counselling models should be adapted and scaled across different climate extremes and care settings.
- International and national professional and health organisations—including the ICN, ANA, and the Ministry of Health of the Republic of Türkiye—should establish concrete mechanisms, such as dedicated funding and continuing professional development programmes, to strengthen nurses’ roles in advocacy, policy development, and research.
- At the workplace level, policies should be developed to protect home-care nurses and field-based healthcare workers exposed to heat stress, including guaranteed rest breaks, appropriate clothing, and adjustments to working hours.
- A broader-scope follow-on review is recommended to extend this search to additional climate hazards (e.g., wildfire smoke, flooding, and other extreme weather events) and chronic disease categories (e.g., cancer, mental health conditions, and neurological disease) that fell outside the deliberate scope boundary of the present review (Section Aim of the Review and Section 5).

In conclusion, the relationship between climate change and chronic diseases is becoming increasingly visible as both a clinical and professional priority for the nursing discipline. However, for this visibility to be translated into sustainable, evidence-based, and actionable nursing practice, the field’s experimental research base must be strengthened, nursing education must be updated accordingly, and nurses must be actively engaged in this process as care providers, advocates, and researchers.

## Figures and Tables

**Table 1:** Keyword-based database search.

Database	Search Date	Search String Used (Abridged)	Number of Results (*n*)
PubMed/MEDLINE	2 June 2026	(“Climate Change”[Mesh] OR “Global Warming”[Mesh] OR “Hot Temperature”[Mesh] OR “Extreme Heat”[Mesh] OR “Air Pollution”[Mesh] OR “climate change”[tiab] OR “global warming”[tiab] OR “heat wave”[tiab] OR heatwave[tiab] OR “extreme heat”[tiab] OR “ambient temperature”[tiab] OR “high temperature”[tiab]) AND (“Chronic Disease”[Mesh] OR “Noncommunicable Diseases”[Mesh] OR “Pulmonary Disease, Chronic Obstructive”[Mesh] OR “Diabetes Mellitus”[Mesh] OR “Cardiovascular Diseases”[Mesh] OR “Heart Failure”[Mesh] OR “Renal Insufficiency, Chronic”[Mesh] OR “chronic disease *”[tiab] OR “chronic illness”[tiab] OR COPD[tiab] OR diabetes[tiab] OR “heart failure”[tiab] OR “chronic kidney disease”[tiab]) AND (“Nursing”[Mesh] OR “Nurses”[Mesh] OR “Nursing Care”[Mesh] OR nurse[tiab] OR nurses[tiab] OR nursing[tiab] OR “patient education”[tiab] OR “self-management”[tiab])	*n* = 121
CINAHL	6 June 2026	((MH “Climate Change+”) OR (MH “Global Warming”) OR (MH “Heat”) OR (MH “Air Pollution”) OR TI(“climate change” OR “global warming” OR “heat wave” OR heatwave OR “extreme heat” OR “ambient temperature” OR “high temperature” OR “air pollution”) OR AB(“climate change” OR “global warming” OR “heat wave” OR heatwave OR “extreme heat” OR “air pollution”)) AND ((MH “Chronic Disease”) OR (MH “Diabetes Mellitus+”) OR (MH “Pulmonary Disease, Chronic Obstructive”) OR (MH “Cardiovascular Diseases+”) OR (MH “Heart Failure”) OR (MH “Kidney Failure, Chronic”) OR TI(“chronic disease *” OR “chronic illness” OR “noncommunicable disease *” OR COPD OR diabetes OR “cardiovascular disease *” OR “heart failure” OR “chronic kidney disease”) OR AB(“chronic disease *” OR “chronic illness” OR COPD OR diabetes OR “heart failure” OR “chronic kidney disease”)) AND ((MH “Nursing Care+”) OR (MH “Nurses+”) OR (MH “Nursing Practice”) OR TI(nurse * OR nursing OR “patient education” OR “self-management”) OR AB(nurse * OR nursing OR “patient education” OR “self-management”))	*n* = 124
Scopus	6 June 2026	TITLE-ABS-KEY(“climate change” OR “global warming” OR “heat wave” OR heatwave OR “extreme heat” OR “ambient temperature” OR “high temperature” OR “air pollution”) AND TITLE-ABS-KEY(“chronic disease *” OR “chronic illness” OR “noncommunicable disease *” OR COPD OR diabetes OR “cardiovascular disease *” OR “heart failure” OR “chronic kidney disease”) AND TITLE-ABS-KEY(nurse OR nurses OR nursing OR “nursing care” OR “patient education” OR “self-management”)	*n* = 303
Web of Science	19 June 2026	TS = (“climate change” OR “global warming” OR “heat wave” OR heatwave OR “extreme heat” OR “ambient temperature” OR “high temperature” OR “air pollution”) AND TS = (“chronic disease” OR “chronic diseases” OR “chronic illness” OR “noncommunicable disease” OR “noncommunicable diseases” OR COPD OR diabetes OR “cardiovascular disease” OR “cardiovascular diseases” OR “heart failure” OR “chronic kidney disease”) AND TS = (nurse OR nurses OR nursing OR “nursing care” OR “patient education” OR “self-management”)	*n* = 176
Cochrane Library	19 June 2026	#1 [mh “Climate Change”] #2 [mh “Hot Temperature”] #3 [mh “Air Pollution”] #4 (“climate change” OR “global warming” OR “heat wave” OR heatwave OR “extreme heat” OR “ambient temperature” OR “high temperature” OR “air pollution”):ti,ab,kw #5 {OR #1-#4} #6 [mh “Chronic Disease”] #7 [mh “Noncommunicable Diseases”] #8 [mh “Pulmonary Disease, Chronic Obstructive”] #9 [mh “Diabetes Mellitus”] #10 [mh “Cardiovascular Diseases”] #11 [mh “Heart Failure”] #12 [mh “Renal Insufficiency, Chronic”] #13 (“chronic disease *” OR “chronic illness” OR “noncommunicable disease *” OR COPD OR diabetes OR “cardiovascular disease *” OR “heart failure” OR “chronic kidney disease”):ti,ab,kw #14 {OR #6-#13} #15 [mh “Nursing”] #16 [mh “Nurses”] #17 [mh “Nursing Care”] #18 (nurse * OR nursing OR “patient education” OR “self-management”):ti,ab,kw #19 {OR #15-#18} #20 #5 AND #14 AND #19	*n* = 2
TR Dizin	22 June 2026	İklim + kronik hastalık ekseni: iklim değişikliği + kronik hastalık; küresel ısınma + kronik hastalık; sıcak hava dalgası + kronik. İklim + hemşirelik ekseni: iklim değişikliği + hemşire; küresel ısınma + hemşirelik; climate change + nursing	*n* = 128
DergiPark	22 June 2026	The first 100 records ranked by relevance were downloaded for screening. TR Dizin served as the primary source for the Turkish-language literature. Because the DergiPark search engine does not support field or phrase operators and substantially overlaps with TR Dizin, it was used only as a supplementary source through a manual review of results ranked by relevance.	*n* = 100
Council of Higher Education (YÖK) National Thesis Center	22 June 2026	All fields: “iklim” AND “kronik” = 0; All fields: “küresel ısınma” AND “hemşire” = 0; All fields: “sıcak hava dalgası” = 0; All fields: “climate change” AND “nursing” = 3	*n* = 3
Total			*n* = 957

Note: * = truncation symbol, capturing word variants (e.g., disease/diseases). MeSH: Medical Subject Headings; tiab: title/abstract field (PubMed); MH: major heading (CINAHL); TI: title; AB: abstract; TS: topic search (Web of Science). The searches were conducted between 2 and 22 June 2026. The DergiPark search was restricted to the first 100 relevance-ranked records because the platform does not support field-restricted or phrase-level querying and its holdings substantially overlap with TR Dizin; this threshold was applied post hoc and was not pre-specified in the OSF protocol, a deviation reported transparently here (see Section 5). Backward and forward citation searching of the included sources was not performed and is recommended for future updates of this review.

**Table 2:** PCC inclusion criteria.

Component	Definition
Population	Adults (≥18 years) with one of the major chronic non-communicable diseases targeted by the search strategy (Table 1): chronic obstructive pulmonary disease (COPD)/chronic respiratory diseases, diabetes mellitus, cardiovascular diseases (including heart failure and ischaemic heart disease), chronic kidney disease, or hypertension. Sources whose population was nurses or other clinicians (e.g., knowledge, attitude, or preparedness studies) or the health-system/environmental-impact level were also eligible when they addressed the Concept below; these are reported and interpreted separately from patient-focused sources throughout Section 3 and Section 4. Sources addressing pregnancy-related manifestations of the target diseases above—specifically gestational diabetes mellitus and pre-eclampsia/hypertensive disorders of pregnancy—were also eligible as disease-specific instances of the diabetes mellitus and cardiovascular/hypertensive disease categories, respectively, even where the immediate study population was pregnant women rather than the general adult population; broader maternal or neonatal outcomes without a named target-disease link were not eligible under this criterion. Sources centred on populations outside this scope (e.g., paediatric or general-population sources without a named adult chronic disease, or maternal/neonatal outcomes without a qualifying target-disease link as defined above) were excluded under E5.
Concept	The effects of exposure to global warming and climate change-related hazards, including extreme heat, extreme cold, air pollution, greenhouse gas emissions, and vector-borne diseases, on the course of chronic diseases, morbidity, and mortality, together with nursing care addressing these effects, nurse-led interventions, patient education, self-management, and nurses’ awareness and knowledge levels.
Context	Healthcare settings in which nursing care, nursing roles, nursing interventions, or the nursing discipline were addressed, including hospitals, primary care settings, and community-based care. No geographical restrictions were applied.

**Table 3:** Characteristics of the included sources (*n* = 23).

No	Author(s) (Year)	Country	Design	Disease Category	Climate-Related Risk
1	Arslan et al. [11]	The Netherlands	Cross-sectional/Survey	Respiratory System Diseases	Greenhouse Gas Emissions
2	Akbaş & Erol [12]	Turkey	Review	Multisystem Conditions	General/Multiple
3	Sebastião et al. [13]	Portugal	Review	Respiratory System Diseases	Air Pollution
4	Nicholas et al. [14]	United States	Case + Review	Rheumatological/Infectious Diseases	Vector-Borne
5	Scullion [15]	United Kingdom	Review	Respiratory System Diseases	Greenhouse Gas Emissions
6	Hodges & Al Hodges [16]	United States	Case + Review	Multisystem Conditions	Extreme Heat
7	Gumabay et al. [17]	The Philippines	Qualitative	Cardiovascular System Diseases	Extreme Cold
8	Squires [18]	United Kingdom	Review	Respiratory System Diseases	General/Multiple
9	Asal et al. [19]	Egypt/Saudi Arabia	Cross-Sectional	Kidney Diseases	Greenhouse Gas Emissions
10	Chaseling et al. [20]	Australia	Cross-sectional/Survey	Cardiovascular System Diseases	Extreme Heat
11	Mehta et al. [21]	United States	Cohort Study	Mental Health Disorders	Extreme Heat
12	Frantzen & Kaifie [22]	Germany	Cross-sectional/Survey	Multisystem Conditions	Extreme Heat
13	Portela Dos Santos et al. [23]	Switzerland	Pilot Cohort Study	Cardiovascular System Diseases	Extreme Cold/Air Pollution
14	George et al. [24]	United States	Review	Respiratory System Diseases	General/Multiple
15	Jehn et al. [25]	Germany	Randomised Controlled Trial (RCT)	Respiratory System Diseases	Extreme Heat
16	Carrier & Padilla [26]	United States	Review	Kidney Diseases	Extreme Heat
17	Dönmez & Kurt [27]	Turkey	Review	Women’s Health/Neonatal Conditions	General/Multiple
18	Stewart et al. [28]	Australia	Randomised Controlled Trial (RCT)	Cardiovascular System Diseases	General/Multiple
19	Ziegler et al. [29]	United States	Review	Multisystem Conditions	General/Multiple
20	Stewart et al. [30]	Australia	Randomised Controlled Trial (Baseline)	Cardiovascular System Diseases	General/Multiple
21	Genç & Akarsu [31]	Turkey	Review	Multisystem Conditions	General/Multiple
22	Kes [3]	Turkey	Bibliometric Study	Multisystem Conditions	General/Multiple
23	Padilla & Sabol [32]	United States	Review	Metabolic Diseases	Extreme Heat/Air Pollution

**Table 4:** Distribution by disease category.

Disease Category	*n*	%
Respiratory Diseases	6	26.1%
Multisystem Conditions	6	26.1%
Cardiovascular Diseases	5	21.7%
Kidney Diseases	2	8.7%
Mental Health Conditions	1	4.3%
Metabolic Diseases	1	4.3%
Rheumatological/Infectious Conditions	1	4.3%
Women’s Health/Neonatal Outcomes	1	4.3%
Total	23	100%

**Table 5:** Distribution by type of climate-related risk.

Climate-Related Risk Type	*n*	%
General/Multiple Climate Change	9	39.1%
Extreme Heat	6	26.1%
Greenhouse Gas Emissions/Carbon Footprint	3	13.0%
Extreme Cold	1	4.3%
Extreme Cold + Air Pollution	1	4.3%
Air Pollution	1	4.3%
Vector-Borne	1	4.3%
Extreme Heat + Air Pollution	1	4.3%
Total	23	100%

**Table 6:** Distribution by study design.

Study Design	*n*	%
Review (Narrative/Position Paper)	10	43.5%
Cross-Sectional Study/Survey	4	17.4%
Randomised Controlled Trial (RCT)	2	8.7%
RCT (Baseline Characteristics Report)	1	4.3%
Cohort Study	1	4.3%
Pilot Cohort Study	1	4.3%
Qualitative	1	4.3%
Case Report + Review	2	8.7%
Bibliometric Analysis	1	4.3%
Total	23	100%

**Table 7:** Distribution by country.

Country	*n*	%
United States	7	30.4%
Türkiye	4	17.4%
Australia	3	13.0%
United Kingdom	2	8.7%
Germany	2	8.7%
The Netherlands	1	4.3%
Portugal	1	4.3%
The Philippines	1	4.3%
Egypt/Saudi Arabia	1	4.3%
Switzerland	1	4.3%
Total	23	100%

**Table 8:** Distribution of nursing intervention themes. Operational definitions for each theme are provided in the added column below.

Theme	Operational Definition	*n*	%	Relevant Sources
Patient/Family Education and Counselling	Nursing content in which patients and/or family members were given information, skills training, or counselling to support self-management or protective behaviour in relation to a climate-related hazard (e.g., heat-action planning, inhaler technique, air-quality alerts, disaster preparedness).	17	73.9	[7,8,9,10,11,12,13,15,17,19,21,22,23,26,27,28,29]
Environmental Risk Assessment/Screening	Nursing content describing systematic assessment of a patient’s or population’s exposure or vulnerability to a climate-related hazard (e.g., occupational exposure, heat stress, air pollution, socioeconomic vulnerability).	15	65.2	[8,9,10,12,15,16,19,20,21,23,25,26,27,28,29]
Advocacy	Nursing content describing efforts to promote environmental justice, sustainable/green prescribing, or climate-mitigation policy on behalf of patients or communities.	9	39.1	[12,15,16,22,26,27,29,31,32]
Policy Development/Sustainable and Green Practices	Nursing content describing institutional or practice-level sustainability initiatives (e.g., reducing carbon footprint, low-global-warming-potential inhaler selection, green nephrology practices) or formal policy-development activity.	7	30.4	[7,8,9,11,16,21,24]
Disaster/Emergency Preparedness	Nursing content describing planning for, or response to, acute climate-related emergencies (e.g., heatwave, extreme-weather event) at the patient, service, or system level.	6	26.1	[19,25,26,27,28,29]
Care Coordination/Referral/Case Management	Nursing content describing coordination of care across settings or providers, or case-management activity, in relation to a climate-related hazard.	5	21.7	[14,16,20,28,30]
Evidence Generation/Research Role	Nursing content describing the nurse’s role as a generator of research evidence on the climate–chronic disease relationship, rather than solely as a care provider.	4	17.4	[3,20,21,26]
Self-Management Support	Nursing content describing support for patients’ independent, ongoing adjustment of behaviour or treatment in response to a climate-related trigger; distinguished from Education/Counselling by its focus on the patient’s own self-regulation rather than one-off instruction.	3	13.0	[15,17,25]
Monitoring/Surveillance/Telehealth	Nursing content describing remote or technology-supported monitoring or surveillance of patient status in relation to a climate-related hazard.	3	13.0	[13,23,25]
Integration into Nursing Education Curricula	Nursing content describing the incorporation, proposal, or evaluation of climate–health content within formal undergraduate or postgraduate nursing education.	3	13.0	[3,18,23]
Leadership/Multifaceted Professional Model (ICN/ANA)	Nursing content explicitly framed through a multifaceted professional-identity model (ICN or ANA), encompassing educator, advocate, change-agent, leader, caregiver, and supervisor roles collectively rather than any single function.	2	8.7	[27,32]
Home Visit–Based Care Coordination Model	Nursing content describing a specific, named model of nurse-coordinated, multicomponent home-visit care (the RESILIENCE model).	2	8.7	[28,30]

## Data Availability

The data extracted and synthesised for this scoping review are available within the article and as Supplementary Materials, comprising: (i) the complete data-extraction form for all included sources (Table S2; provided in English), covering all fields listed in Section 2.5; (ii) the synthesis tables underlying Table 3, Table 4, Table 5, Table 6, Table 7 and Table 8 of this manuscript; and (iii) the PRISMA-ScR record-selection counts underlying Figure 1 and Section 2.4, including the E1–E5 exclusion breakdown. Database export files and deduplication logs (Zotero, Rayyan) generated during screening are archived by the author and available from the corresponding author upon reasonable request. Further inquiries can be directed to the corresponding author.

## Supplementary Materials

The following supporting information can be downloaded at https://www.mdpi.com/article/10.3390/healthcare14183044/s1: Table S1: PRISMA-ScR Checklist; Table S2: Data extraction form for the 23 included sources (data_extraction_form_23_studies_EN.xlsx).

## Abbreviations

The following abbreviations are used in this manuscript:

ACE	Angiotensin-Converting Enzyme
ANA	American Nurses Association
CI	Confidence Interval
COPD	Chronic Obstructive Pulmonary Disease
FEV_1_	Forced Expiratory Volume in 1 Second
GOLD	Global Initiative for Chronic Obstructive Lung Disease
GWP	Global Warming Potential
HDI	Human Development Index
HR	Hazard Ratio
ICN	International Council of Nurses
JBI	Joanna Briggs Institute
OSF	Open Science Framework
PCC	Population–Concept–Context
PRISMA-ScR	Preferred Reporting Items for Systematic Reviews and Meta-Analyses Extension for Scoping Reviews
RCT	Randomised Controlled Trial
YÖK	Council of Higher Education (Yükseköğretim Kurulu, Türkiye)
