# Supplementary material for: Climate Change and Nursing Care in Major Chronic Diseases: A Scoping Review of Nursing Perspectives and Roles
Source: Healthcare (Basel). 2026 Sep 16;14(18):3044. doi: 10.3390/healthcare14183044 (PMC13606497; doi:10.3390/healthcare14183044)
Supplement: Supplementary file 1 [file healthcare-14-03044-s001.zip › Table S1. PRISMA-ScR_checklist.pdf]

PRISMA 2020 Checklist

Section and Topic	Item #	Checklist item	Location where item is reported
**TITLE**			
Title	1	Identify the report as a systematic review.	Title page (title identifies the study as 'A Scoping Review')
**ABSTRACT**			
Abstract	2	See the PRISMA 2020 for Abstracts checklist.	Abstract (structured: Background/Objectives, Methods, Results, Conclusions)
**INTRODUCTION**			
Rationale	3	Describe the rationale for the review in the context of existing knowledge.	Section 1, Introduction, para. 1–2
Objectives	4	Provide an explicit statement of the objective(s) or question(s) the review addresses.	Section 1.1, Aim of the Review
**METHODS**			
Eligibility criteria	5	Specify the inclusion and exclusion criteria for the review and how studies were grouped for the syntheses.	Section 2.3, Eligibility Criteria; Table 1 (PCC framework); exclusion codes E1–E5
Information sources	6	Specify all databases, registers, websites, organisations, reference lists and other sources searched or consulted to identify studies. Specify the date when each source was last searched or consulted.	Section 2.2, Identification of Relevant Studies; Table 2
Search strategy	7	Present the full search strategies for all databases, registers and websites, including any filters and limits used.	Table 2 (full search strings per database)
Selection process	8	Specify the methods used to decide whether a study met the inclusion criteria of the review, including how many reviewers screened each record and each report retrieved, whether they worked independently, and if applicable, details of automation tools used in the process.	Section 2.4, Record Selection Process – NOTE: number/independence of reviewers not stated in Methods (only in Limitations); add explicit statement
Data collection process	9	Specify the methods used to collect data from reports, including how many reviewers collected data from each report, whether they worked independently, any processes for obtaining or confirming data from study investigators, and if applicable, details of automation tools used in the process.	Section 2.5, Data Extraction – NOTE: number/independence of reviewers not stated in Methods (only in Limitations); add explicit statement
Data items	10a	List and define all outcomes for which data were sought. Specify whether all results that were compatible with each outcome domain in each study were sought (e.g. for all measures, time points, analyses), and if not, the methods used to decide which results to collect.	Section 2.5, Data Extraction; Appendix A

PRISMA 2020 Checklist

Section and Topic	Item #	Checklist item	Location where item is reported
	10b	List and define all other variables for which data were sought (e.g. participant and intervention characteristics, funding sources). Describe any assumptions made about any missing or unclear information.	Section 2.5, Data Extraction; Appendix A
Study risk of bias assessment	11	Specify the methods used to assess risk of bias in the included studies, including details of the tool(s) used, how many reviewers assessed each study and whether they worked independently, and if applicable, details of automation tools used in the process.	N/A – not applicable to scoping reviews; Section 2.6 describes the descriptive level-of-evidence classification used instead
Effect measures	12	Specify for each outcome the effect measure(s) (e.g. risk ratio, mean difference) used in the synthesis or presentation of results.	N/A – no meta-analysis performed; narrative synthesis only (Section 2.7)
Synthesis methods	13a	Describe the processes used to decide which studies were eligible for each synthesis (e.g. tabulating the study intervention characteristics and comparing against the planned groups for each synthesis (item #5)).	Section 2.7, Data Synthesis
	13b	Describe any methods required to prepare the data for presentation or synthesis, such as handling of missing summary statistics, or data conversions.	N/A – no data conversion or pooling performed
	13c	Describe any methods used to tabulate or visually display results of individual studies and syntheses.	Tables 3–8; Section 3
	13d	Describe any methods used to synthesize results and provide a rationale for the choice(s). If meta-analysis was performed, describe the model(s), method(s) to identify the presence and extent of statistical heterogeneity, and software package(s) used.	Section 2.7, Data Synthesis (descriptive/narrative synthesis; no meta-analysis)
	13e	Describe any methods used to explore possible causes of heterogeneity among study results (e.g. subgroup analysis, meta-regression).	N/A – no meta-analysis performed
	13f	Describe any sensitivity analyses conducted to assess robustness of the synthesized results.	N/A – no meta-analysis performed
Reporting bias assessment	14	Describe any methods used to assess risk of bias due to missing results in a synthesis (arising from reporting biases).	N/A – not assessed; not applicable to scoping review methodology (acknowledged in Section 5, Limitations)
Certainty assessment	15	Describe any methods used to assess certainty (or confidence) in the body of evidence for an outcome.	N/A – not assessed (acknowledged in Section 5, Limitations)
**RESULTS**			
Study selection	16a	Describe the results of the search and selection process, from the number of records identified in the search to the number of studies included in the review, ideally using a flow diagram.	Section 3.1; Figure 1 (PRISMA-ScR flow diagram)
	16b	Cite studies that might appear to meet the inclusion criteria, but which were excluded, and explain why they were excluded.	Partially reported – aggregate exclusion

Section and Topic	Item #	Checklist item	Location where item is reported
			counts by category (E1–E5) are given in Section 2.4 and Figure 1; individual citations for excluded studies are not listed
Study characteristics	17	Cite each included study and present its characteristics.	Section 3.2; Table 3
Risk of bias in studies	18	Present assessments of risk of bias for each included study.	N/A – scoping review; Table 7 presents the descriptive level-of-evidence classification instead
Results of individual studies	19	For all outcomes, present, for each study: (a) summary statistics for each group (where appropriate) and (b) an effect estimate and its precision (e.g. confidence/credible interval), ideally using structured tables or plots.	Table 3 (level of evidence per study); Appendix A (full extraction form); no effect estimates – not applicable to scoping review
Results of syntheses	20a	For each synthesis, briefly summarise the characteristics and risk of bias among contributing studies.	Sections 3.3–3.9; Tables 4–8
	20b	Present results of all statistical syntheses conducted. If meta-analysis was done, present for each the summary estimate and its precision (e.g. confidence/credible interval) and measures of statistical heterogeneity. If comparing groups, describe the direction of the effect.	N/A – no meta-analysis performed
	20c	Present results of all investigations of possible causes of heterogeneity among study results.	N/A – no meta-analysis performed
	20d	Present results of all sensitivity analyses conducted to assess the robustness of the synthesized results.	N/A – no meta-analysis performed
Reporting biases	21	Present assessments of risk of bias due to missing results (arising from reporting biases) for each synthesis assessed.	N/A – not assessed
Certainty of evidence	22	Present assessments of certainty (or confidence) in the body of evidence for each outcome assessed.	N/A – not assessed
**DISCUSSION**			
Discussion	23a	Provide a general interpretation of the results in the context of other evidence.	Section 4, Discussion
	23b	Discuss any limitations of the evidence included in the review.	Sections 4 and 5 (Limitations)
	23c	Discuss any limitations of the review processes used.	Section 5, Limitations
	23d	Discuss implications of the results for practice, policy, and future research.	Section 6, Conclusions and Recommendations
**OTHER INFORMATION**			
Registration and	24a	Provide registration information for the review, including register name and registration number, or state that the review was not	Section 2.1, Protocol

PRISMA 2020 Checklist

Section and Topic	Item #	Checklist item	Location where item is reported
protocol		registered.	and Registration (OSF, osf.io/j7bfy – registered retrospectively)
	24b	Indicate where the review protocol can be accessed, or state that a protocol was not prepared.	Section 2.1, Protocol and Registration (protocol accessible via the OSF registration link)
	24c	Describe and explain any amendments to information provided at registration or in the protocol.	NOT STATED – add an explicit sentence, e.g. 'No amendments were made to the registered protocol.'
Support	25	Describe sources of financial or non-financial support for the review, and the role of the funders or sponsors in the review.	End matter, Funding
Competing interests	26	Declare any competing interests of review authors.	End matter, Conflicts of Interest
Availability of data, code and other materials	27	Report which of the following are publicly available and where they can be found: template data collection forms; data extracted from included studies; data used for all analyses; analytic code; any other materials used in the review.	End matter, Data Availability Statement; Appendix A (data extraction form provided as a separate Excel file)

From: Page MJ, McKenzie JE, Bossuyt PM, Boutron I, Hoffmann TC, Mulrow CD, et al. The PRISMA 2020 statement: an updated guideline for reporting systematic reviews. BMJ 2021;372:n71. doi: 10.1136/bmj.n71.
This work is licensed under CC BY 4.0. To view a copy of this license, visit https://creativecommons.org/licenses/by/4.0/
